# ^18^F-Fludeoxyglucose PET/CT in SCLC: Analysis of the CONVERT Randomized Controlled Trial

**DOI:** 10.1016/j.jtho.2019.03.023

**Published:** 2019-07

**Authors:** Prakash Manoharan, Ahmed Salem, Hitesh Mistry, Michael Gornall, Susan Harden, Peter Julyan, Imogen Locke, Jonathan McAleese, Rhona McMenemin, Nazia Mohammed, Michael Snee, Sarah Woods, Thomas Westwood, Corinne Faivre-Finn

**Affiliations:** a Department of Radiology, The Christie National Health Service Foundation Trust, Manchester, United Kingdom; b Department of Nuclear Medicine, The Christie National Health Service Foundation Trust, Manchester, United Kingdom; c Division of Cancer Sciences, University of Manchester, Manchester, United Kingdom; d Department of Clinical Oncology, The Christie National Health Service Foundation Trust, Manchester, United Kingdom; e Division of Pharmacy, University of Manchester, Manchester, United Kingdom; f Department of Clinical Oncology, Addenbrooke’s Hospital, Cambridge, United Kingdom; g Department of Clinical Oncology, The Royal Marsden National Health Service Foundation Trust, Surrey, United Kingdom; h Department of Clinical Oncology, Northern Ireland Cancer Centre, Belfast City Hospital, Belfast, United Kingdom; i Department of Clinical Oncology, Northern Centre for Cancer Care, Freeman Hospital, Newcastle-upon-Tyne, United Kingdom; j Department of Clinical Oncology, Beatson West of Scotland Cancer Centre, Glasgow, United Kingdom; k Department of Clinical Oncology, Leeds Cancer Centre, St James’s Hospital, Leeds, United Kingdom; l Department of Nuclear Medicine, Manchester University National Health Service Foundation Trust, Manchester, United Kingdom

**Keywords:** small-cell, lung cancer, ^18^F-FDG PET/CT, staging, survival

## Abstract

**Introduction:**

We used phase-3 CONVERT trial data to investigate the impact of fludeoxyglucose F 18 (^18^F-FDG) positron emission tomography (PET)/computed tomography (CT) in SCLC.

**Methods:**

CONVERT randomized patients with limited-stage SCLC to twice-daily (45 Gy in 30 fractions) or once-daily (66 Gy in 33 fractions) chemoradiotherapy. Patients were divided into two groups in this unplanned analysis: those staged with conventional imaging (contrast-enhanced thorax and abdomen CT and brain imaging with or without bone scintigraphy) and those staged with ^18^F-FDG PET/CT in addition.

**Results:**

Data on a total of 540 patients were analyzed. Compared with patients who underwent conventional imaging (n = 231), patients also staged with ^18^F-FDG PET/CT (n = 309) had a smaller gross tumor volume (*p* = 0.003), were less likely to have an increased pretreatment serum lactate dehydrogenase level (*p* = 0.035), and received more chemotherapy (*p* = 0.026). There were no significant differences in overall (hazard ratio = 0.87, 95% confidence interval: 0.70–1.08, *p* = 0.192) and progression-free survival (hazard ratio = 0.87, 95% confidence interval: 0.71–1.07], *p* = 0.198) between patients staged with or without ^18^F-FDG PET/CT. In the conventional imaging group, we found no survival difference between patients staged with or without bone scintigraphy. Although there were no differences in delivered radiotherapy dose, ^18^F-FDG PET/CT–staged patients received lower normal tissue (lung, heart, and esophagus) radiation doses. Apart from a higher incidence of late esophagitis in patients staged with conventional imaging (for grade ≥1, 19% versus 11%; [*p* = 0.012]), the incidence of acute and late radiotherapy-related toxicities was not different between the two groups.

**Conclusion:**

In CONVERT, survival outcomes were not significantly different in patients staged with or without ^18^F-FDG PET/CT. However, this analysis cannot support the use or omission of ^18^F-FDG PET/CT owing to study limitations.

## Introduction

Lung cancer is the leading cause of cancer mortality worldwide.^1^ SCLC constitutes 13% of lung cancer cases in developed countries.^2^ Survival of SCLC is poor, with modest improvements over the past three decades^2^ mainly thanks to advancements in scheduling. The U.S. Food and Drug Administration has not approved any new drugs for the treatment of SCLC since 1996,^3^ highlighting the importance of optimizing combination therapies.

A two-stage classification system is widely utilized in SCLC. Initially devised by the Veterans Administration Lung Cancer Study, this system classifies SCLC into limited- or extensive-stage disease according to whether the tumor is localized to one hemithorax.^4^ In 2009, the Union for International Cancer Control/American Joint Committee on Cancer, based on an analysis by the International Association for the Study of Lung Cancer, recommended the use of TNM staging in SCLC, as it provides additional prognostic information.^5^ This recommendation was recently confirmed in a CONVERT trial subgroup analysis.^6^ Treatment and outcome vary according to stage, highlighting the importance of accurate staging in SCLC to guide therapeutic decisions and provide prognostication. Standard treatment for fit patients with limited-stage SCLC is concurrent chemoradiotherapy and prophylactic cranial irradiation (PCI).^7^, ^8^, ^9^ In extensive-stage SCLC, standard treatment includes chemotherapy followed by optional consolidative palliative thoracic radiotherapy in responders^10^ and PCI^11^ or serial surveillance brain magnetic resonance imaging (MRI).^12^ The addition of atezolizumab to chemotherapy was recently shown to improve survival in the first-line setting.^13^

On the basis of patterns of metastasis,^14^ conventional imaging for suspected or proven limited-staged SCLC includes contrast-enhanced thorax and abdomen computed tomography (CT) and brain imaging (CT or MRI) with or without bone scintigraphy. The role of staging fludeoxyglucose F 18 (^18^F-FDG) positron emission tomography (PET)/CT is uncertain; however, it is widely utilized routinely in developed countries.^15^ Small retrospective and nonrandomized prospective studies have shown that ^18^F-FDG PET or ^18^F-FDG PET/CT improves SCLC staging accuracy, with pretreatment metabolic and volumetric ^18^F-FDG PET parameters providing additional prognostic information.^16^, ^17^, ^18^, ^19^ Oncology practice guidelines (e.g., the National Comprehensive Cancer Network guidelines) now recommend or suggest using ^18^F-FDG PET/CT when staging patients with SCLC.^20^, ^21^, ^22^, ^23^ However, landmark phase 3 trials that established chemoradiotherapy as standard treatment in limited-staged SCLC were performed before the ^18^F-FDG PET/CT era.^24^ It is therefore likely that a proportion of patients treated with concurrent chemoradiotherapy in these studies had undetected metastatic disease on conventional imaging. It is not known whether the outcome of concurrent chemoradiotherapy–treated patients with limited-stage SCLC staged with conventional imaging and concurrently treated with chemotherapy differs from that of patients staged with ^18^F-FDG PET/CT. Furthermore, randomized studies have not been performed to establish the efficacy of ^18^F-FDG PET/CT over conventional imaging in SCLC.

The impact of ^18^F-FDG PET/CT in SCLC management is not clearly defined. We assessed the effect of radiological staging methods on outcome in patients with limited-stage SCLC treated with concurrent chemoradiotherapy in the phase 3 CONVERT trial.

## Material and Methods

### Trial Design and Participants

Detailed trial design and results were previously published.^8^, ^25^ In summary, CONVERT is a multicenter phase III trial that randomly assigned (1:1 using the minimization method) patients with an Eastern Cooperative Oncology Group performance score of 0 to 2 and limited-stage SCLC (the Veterans Administration Lung Cancer Study definition)^4^ to receive either twice-daily (45 Gy in 30 fractions) or once-daily (66 Gy in 33 fractions) radiotherapy starting on day 22 of chemotherapy cycle 1. Chemotherapy consisted of four to six cycles (according to center choice) of cisplatin and etoposide. PCI was offered, if indicated. A radiotherapy quality assurance program was incorporated.^25^ CONVERT is registered with clinicaltrials.gov (identifier: NCT00433563). The full trial protocol can be found in Supplement 1.

Trial participants gave written informed consent, and the study was done according to the Declaration of Helsinki and Good Clinical Practice guidelines. The trial was reviewed in the United Kingdom by the National Research Ethics Service Committee North West–Greater Manchester Central, which granted ethics approval on December 21, 2007 (REC reference 07/H1008/229). The protocol was also approved by the institutional review board or research ethics committee in each country and at each study center.

Clinical follow-up assessments consisted of weekly review until resolution of acute side effects, then thrice monthly until 1 year after randomization and every 6 months thereafter. A thorax and abdomen CT was required at 6 and 12 months after randomization and thereafter as clinically indicated.

### Staging Investigations

Contrast-enhanced thorax and abdomen CT (within 4 weeks before randomization) was mandated for all trial participants. Brain imaging (CT/MRI) was also required. The trial protocol specified that bone scintigraphy was to be performed if there was a specific clinical indication. ^18^F-FDG PET/CT was permitted according to local practice but not mandated. A maximum of one of the following adverse serum biochemical findings was allowed: alkaline phosphatase level more than 1.5 times the upper limit of normal, sodium level lower than the lower limit of normal, and lactate dehydrogenase (LDH) level higher than the upper limit of normal. Tumor and nodal stage were collected at the time of trial entry according to the American Joint Committee on Cancer staging, seventh edition.^26^

### End Points

The primary trial end point was overall survival (defined as time from randomization to death from any cause). Progression-free survival, a predefined secondary trial end point, was defined as time from randomization to first clinical or radiological evidence of progression. Toxicity was assessed by using common terminology criteria for adverse events (version 3.0).^27^

### Subgroup Analysis

All patients in the CONVERT modified intention-to-treat survival analysis with data on staging investigations were included in this exploratory subgroup analysis.

### Statistical Analysis

Baseline and treatment characteristics, acute (defined as those occurring from chemotherapy cycle 1 to 3 months after treatment completion) and late (defined as those between 3 months and 2 years after treatment completion) toxicities, dosimetric radiotherapy parameters, and chemotherapy and radiotherapy compliance for patients staged with and without ^18^F-FDG PET/CT were compared by using the chi-square or Wilcoxon rank sum tests. The prognostic value of demographic, clinical, and imaging covariates was assessed by using a univariate Cox-regression model. Next, multivariate analysis in which all variables were placed within a single model was conducted.

Kaplan-Meier curves were plotted for the two study groups and survival was compared by using the Mantel-Cox version of the log rank test. Patients who did not experience an event at the end of the study were right-censored. The hazard ratios (HRs) with 95% confidence intervals (CIs) together with *p* values were reported. A *p* value less than 0.05 (adjusted to account for significant differences in baseline characteristics between study groups in primary survival analysis) was considered statistically significant.

Additional statistical details can be found in Supplement 2. All statistical analyses were performed with R software (version 3.4) using the survival library (https://www.r-project.org).

## Results

The modified intention-to-treat survival analysis in CONVERT (recruited 547 patients between April 7, 2008, and Nov 29, 2013) included 543 patients, of whom 540 with data on staging investigations were eligible for this analysis (four patients were lost to follow-up). Detailed trial results were previously published.^8^ In summary, survival outcomes were not significantly different between twice-daily and once-daily concurrent chemoradiotherapy with lower than expected acute and late toxicities in both arms. However, this trial was powered to show superiority of once-daily chemoradiotherapy, not equivalence. For this reason, twice-daily chemoradiotherapy should be considered the standard of care in limited-stage SCLC.

**Figure 1:**
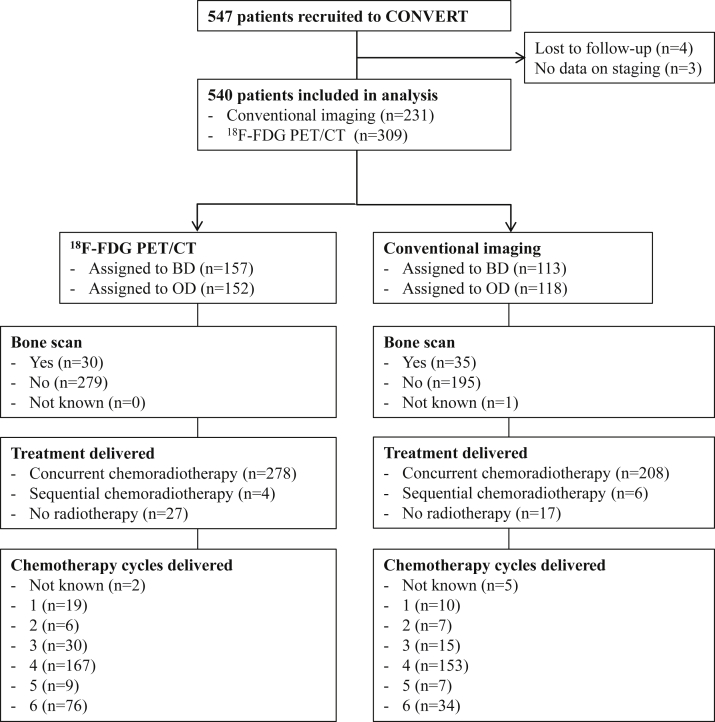
CONSORT diagram. ^18^F-FDG PET/CT, fludeoxyglucose F 18 positron emission tomography–computed tomography; BD, twice daily; OD, once daily.

Of the 540 eligible patients, 231 (43%) underwent staging with conventional imaging (thorax and abdomen CT and brain imaging, with or without bone scintigraphy) and 309 (57%) were staged with ^18^F-FDG PET/CT in addition. A CONSORT diagram for the two study groups is shown in Figure 1.

The utilization of ^18^F-FDG PET/CT was variable in the eight countries recruiting to CONVERT (*p* < 0.001 [Supplementary Table 1]). ^18^F-FDG PET/CT was performed in all patients eligible for this analysis who were recruited in Slovenia and The Netherlands and in 96%, 81%, 76%, 72%, 67%, and 41% of patients recruited in Belgium, Spain, France, Canada, Poland, and the United Kingdom, respectively.

Table 1 shows baseline and treatment characteristics for participants included in this analysis. Compared with patients who underwent conventional imaging, patients who were also staged with ^18^F-FDG PET/CT had a smaller gross tumor volume (73.3 cm^3^ [range 1.6–593] versus 95.7 cm^3^ [range 0.5–635.1], *p* = 0.003), were less likely to have a pretreatment serum LDH level higher than upper limit of normal (20% versus 29% [*p* = 0.035]) and received more chemotherapy (six cycles in 25% versus in 15% [*p* = 0.026]), respectively. There were no other significant differences in baseline (including tumor and nodal staging) and treatment characteristics between the two study groups.

**Table 1:** Baseline and Treatment Characteristics

Characteristic	^18^F-FDG PET/CT and Conventional Imaging (n = 309)	Conventional Imaging (n = 231)	*p* Value
Median age, y (range)	62 (29–84)	62 (36–81)	0.594
Sex			0.204^a^
Male	176 (57%)	118 (51%)	
Female	133 (43%)	113 (49%)	
Ethnicity			0.995^a^
White	299 (97%)	224 (97%)	
African	1 (<1%)	1 (<1%)	
Asian	3 (1%)	2 (1%)	
Other	5 (2%)	4 (2%)	
Not known	1 (<1%)	0 (0%)	
ECOG PS			0.182^a^
0	150 (49%)	98 (42%)	
1	148 (48%)	128 (56%)	
2	11 (3%)	5 (2%)	
Smoking history			0.991^a^
Never-smoker	4 (1%)	3 (1%)	
Former smoker	193 (63%)	143 (62%)	
Current smoker	112 (36%)	85 (37%)	
Adverse biochemical factors			
LDH >ULN	63 (20%)	66 (29%)	**0.035**^a^
Hyponatremia	7 (2%)	4 (2%)	0.899^a^
ALP >1.5× ULN	68 (22%)	41 (18%)	0.267^a^
Radiotherapy			0.723^a^
Once-daily	152 (49%)	118 (51%)	
Twice-daily	157 (51%)	113 (49%)	
UICC/AJCC stage			0.087^a^
I	2 (1%)	2 (1%)	
II	56 (18%)	26 (11%)	
III	233 (75%)	189 (82%)	
Not known	18 (6%)	14 (6%)	
T staging			0.115^a^
T0	6 (2%)	2 (1%)	
T1	42 (14%)	29 (13%)	
T2	105 (34%)	57 (25%)	
T3	60 (19%)	44 (19%)	
T4	84 (27%)	88 (38%)	
Not known	12 (4%)	11 (5%)	
N staging			0.146^a^
N0	53 (17%)	22 (10%)	
N1	38 (12%)	23 (10%)	
N2	160 (52%)	137 (59%)	
N3	48 (16%)	42 (18%)	
Not known	10 (3%)	7 (3%)	
Median gross tumor volume, cm^3^ (range)	73.3 (1.6–593)	95.7 (0.5–635.1)	**0.003**^b^
Bone scan			0.078^a^
Yes	30 (10%)	35 (15%)	
No	279 (90%)	195 (84%)	
Not known	0 (0%)	1 (<1%)	
No. of chemotherapy cycles planned			**0.027**^a^
4	192 (62%)	176 (76%)	
6	117 (38%)	55 (24%)	
No. of chemotherapy cycles given			**0.026**^a^
1	19 (6%)	10 (4%)	
2	6 (2%)	7 (3%)	
3	30 (10%)	15 (6%)	
4	167 (54%)	153 (66%)	
5	9 (3%)	7 (3%)	
6	76 (25%)	34 (15%)	
Not known	2 (<1%)	5 (2%)	
Radiotherapy			0.468^a^
Concurrent	278 (90%)	208 (90%)	
Sequential	4 (1%)	6 (3%)	
No radiotherapy	27 (9%)	17 (7%)	
IMRT			0.172^a^
Yes	53 (17%)	30 (13%)	
No	226 (73%)	185 (80%)	
Not known	30 (10%)	16 (7%)	

*Note:* Boldface indicates statistical significance.

^18^F-FDG PET/CT, fludeoxyglucose F 18 positron emission tomography–computed tomography; ECOG PS, Eastern Cooperative Oncology Group performance status; ALP, alkaline phosphatase; ULN, upper limit of normal; LDH, lactate dehydrogenase; UICC, Union for International Cancer Control; AJCC, American Joint Committee on Cancer; IMRT, intensity-modulated radiation therapy.

a Chi-square test.

b Wilcoxon rank sum test.

**Figure 2:**
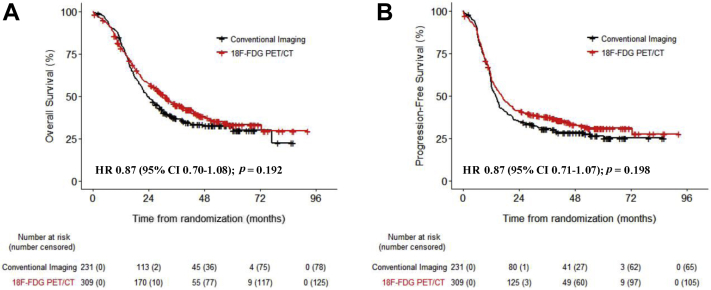
Overall survival and progression-free survival in patients staged with conventional imaging or with fludeoxyglucose F 18 positron emission tomography–computed tomography (18F-FDG PET/CT) in addition. (*A*) Overall survival. (*B*) Progression-free survival. HR, hazard ratio; CI, confidence interval.

**Figure 3:**
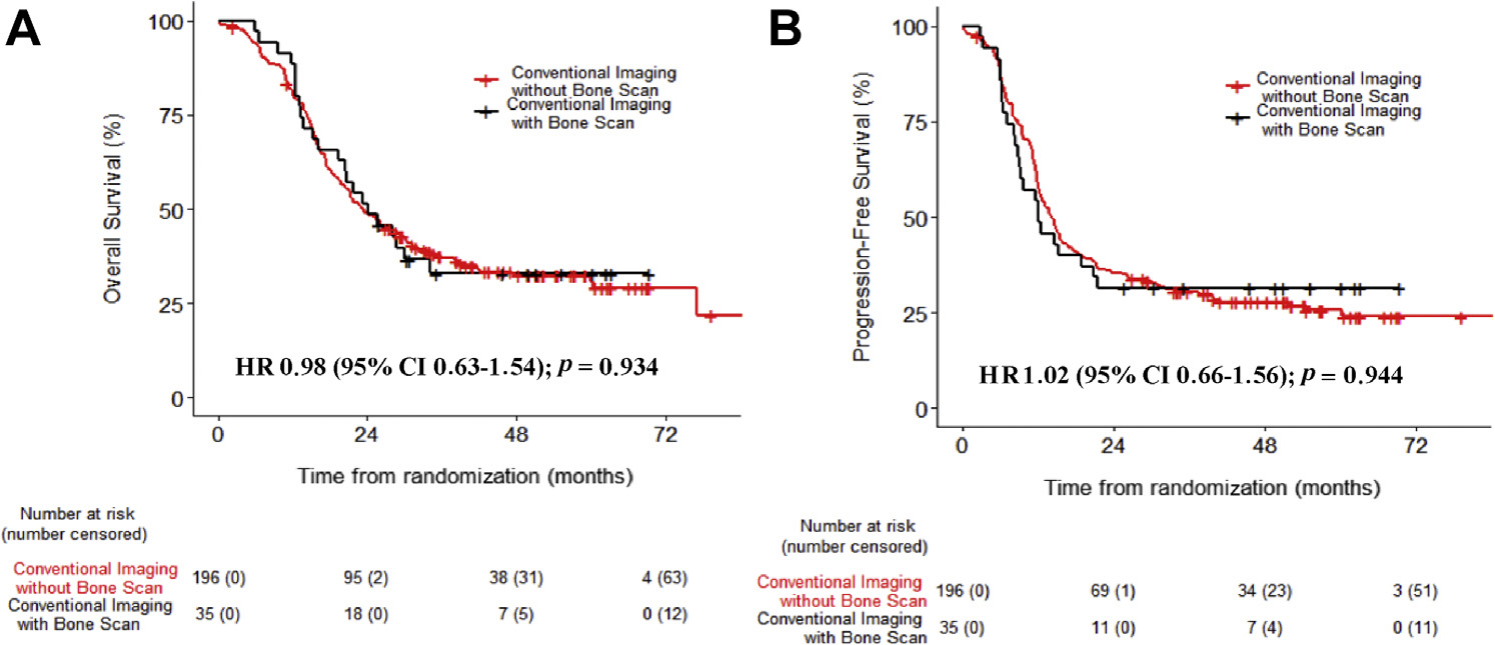
Overall and progression-free survival in patients staged using conventional imaging with or without bone scintigraphy. (*A*) Overall survival. (*B*) Progression-free survival. HR, hazard ratio; CI, confidence interval.

Table 2 and Supplementary Table 2 show the results of the overall and progression-free survival univariate and multivariate analyses, respectively. There were no significant differences in overall (HR = 0.87, 95% CI: 0.70–1.08, unadjusted *p* = 0.192, adjusted *p* = 0.345) and progression-free survival (HR = 0.87, 95% CI 0.71–1.07, unadjusted *p* = 0.198, adjusted *p* = 0.405) between patients staged with or without ^18^F-FDG PET/CT (Fig. 2 and Table 3). These results were observed irrespective of the treatment group (*p* > 0.05) (Supplementary Fig. 1), TNM stage (stage I–II versus III [*p* = 0.543]), or country of recruitment (United Kingdom versus country other than the United Kingdom [*P* > 0.5]). Country-specific subanalyses were not performed because of the small patient numbers. In patients staged by using conventional imaging, we found no significant survival difference between patients who were staged with (n = 35) or without (n = 196) bone scintigraphy (Fig. 3).

**Table 2:** Univariate and Multivariate Overall Survival Analysis

Characteristic	Patients	Univariate Analysis		Multivariate Analysis	
	Events/n	HR (95% CI)	*p* Value	HR (95% CI)	*p* Value
ECOG PS 1 or 2 v 0	337/540	1.38 (1.11–1.72)	0.003	1.29 (0.99–1.65)	0.051
Age	337/540	1.01 (1.00–1.03)	0.060	1.01 (0.99–1.03)	0.226
log (GTV)	288/480	1.37 (1.21–1.55)	<0.001	1.26 (1.08–1.47)	**0.003**
Heart dose (%)	282/469	1.00 (0.99–1.02)	0.668	1.00 (0.99–1.01)	0.544
V20 Lung (%)	300/493	1.03 (1.02–1.05)	<0.001	1.01 (0.99–1.02)	0.223
ALP >1.5 × ULN Yes vs. no	337/540	1.27 (0.57–2.86)	0.556	3.91 (0.90–16.94)	0.069
Hyponatremia Yes vs. no	337/540	0.87 (0.67–1.14)	0.312	0.95 (0.68–1.33)	0.766
LDH >ULN Yes vs. no	337/540	0.92 (0.71–1.18)	0.497	1.00 (0.73–1.37)	0.993
Smoking	337/540				
Ex-smoker vs. never-smoker		0.88 (0.33–2.38)	0.808	1.69 (0.41–6.96)	0.466
Current smoker vs. never-smoker		1.04 (0.38–2.80)	0.946	1.93 (0.47–7.97)	0.364
Weight loss >10% Yes vs. no	316/500	1.87 (1.16–3.02)	0.010	2.02 (1.16–3.53)	**0.013**
FEV1, % predicted	320/515	1.00 (0.99–1.00)	0.248	0.99 (0.99–1.00)	0.108
KCO, % predicted	320/515	1.00 (1.00–1.00)	0.166	1.00 (0.99–1.00)	0.536
Disease stage III vs. I or II	323/509	1.69 (1.22–2.34)	0.001	1.25 (0.85–1.83)	0.262
^18^F-FDG PET/CT Yes vs. no	337/540	0.87 (0.70–1.08)	0.192	0.98 (0.76–1.26)	0.865

*Note:* Boldface indicates statistical significance.

HR, hazard ratio; CI, confidence interval; ECOG PS, Eastern Cooperative Oncology Group performance status; GTV, gross tumor volume; V20 lung, V20 proportion of the lung receiving 20 Gy; ALP, alkaline phosphatase; ULN, upper limit of normal; FEV1, forced expiratory volume in 1 second; LDH, lactate dehydrogenase; KCO, Krogh transfer factor; ^18^F-FDG PET/CT, fludeoxyglucose F 18 positron emission tomography–computed tomography.

**Table 3:** Comparison of Overall and Progression-Free Survival between the Two Groups

Outcome	^18^F-FDG PET/CT and Conventional Imaging	Conventional Imaging	Log Rank (*p* Value)
Overall survival			
Hazard ratio = 0.87 (95% CI: 0.70–1.08)			
Median	31 mo (26–39)	23 mos (21–29)	0.192
1-y	79% (74–84)	82% (77–87)	
2-y	57% (52–63)	49% (43–56)	
Progression-free survival			
Hazard ratio: 0.87 (95% CI 0.71–1.07)			
Median	17 mo (14–20)	14 mo (12–16)	0.198
1-y	61% (56–67)	58% (52–65)	
2-y	41% (36–47)	35% (29–42)	

^18^F-FDG PET/CT, fludeoxyglucose F 18 positron emission tomography–computed tomography; CI, confidence interval; NR, not reached.

Supplementary Table 3 lists the sites of tumor progression in patients staged with or without ^18^F-FDG PET/CT. We also analyzed patient survival following progression in the two groups. There was no significant difference in overall survival after radiological progression in patients staged by using conventional imaging (median 5.9 months [95% CI: 4.4–7.2]) compared with in those staged by using ^18^F-FDG PET/CT in addition (median 5.8 months [95% CI: 4.9–7.1]) (*p* = 0.945).

There were no significant differences in the delivered radiotherapy dose and the optimal number of delivered radiotherapy fractions, as defined per protocol (30 fractions in the twice-daily arm and 33 fractions in the once-daily arm)^25^ in patients staged with or without ^18^F-FDG PET/CT (*p* > 0.05) (Supplementary Table 4).

Patients staged with ^18^F-FDG PET/CT received a lower normal tissue (lung, heart, and esophageal) radiation dose than did patients staged by using conventional imaging (Supplementary Table 5).

The incidences of acute and late radiotherapy-related toxicities were not different between the two groups apart from a significantly higher incidence of late esophagitis in patients staged by using conventional imaging compared with in patients staged by using ^18^F-FDG PET/CT in addition (for grade ≥1, 19% versus 11% [*p* = 0.012]) (Supplementary Tables 6 and 7).

## Discussion

In this CONVERT subgroup analysis we found that survival outcomes were not significantly different in patients with limited-stage SCLC staged with or without ^18^F-FDG PET/CT. The role of ^18^F-FDG PET/CT in the staging and selection of patients with SCLC for concurrent chemoradiotherapy is uncertain owing to the paucity of robust data. This unplanned analysis provides hypothesis-generating evidence within a randomized controlled trial on the role of radiological staging investigations in selecting patients with SCLC for concurrent chemoradiotherapy. Our findings also suggest that the better than expected outcome in both arms of the CONVERT trial (compared with the outcomes of previous landmark studies^9^) is not explained by the use of ^18^F-FDG PET/CT but is likely the result of modern radiotherapy, supportive care, and salvage therapy improvements^8^ or a consequence of the CONVERT trial eligibility criteria (e.g., exclusion of patients with adverse biochemical features). In patients staged by using conventional imaging, we found no survival difference between patients staged with or without bone scintigraphy. Although we acknowledge the small number of patients included in this comparison, these results question the merit of bone scintigraphy in the staging of SCLC.

The glucose analogue FDG reflects intracellular glucose metabolism, which is increased in tumors, including lung cancer. In the past two decades, ^18^F-FDG PET/CT has emerged as an important oncological staging modality and is now well established in NSCLC as being superior to CT alone in detecting lymph node and distant metastases.^28^ In these initial validation studies, pathological confirmation of metastatic disease detected by ^18^F-FDG PET or ^18^F-FDG PET/CT was almost always obtained. It is now recognized that adding ^18^F-FDG PET or ^18^F-FDG PET/CT to the NSCLC diagnostic algorithm will result in a change in the treatment decision and/or treatment intent (cure versus palliation) in some patients. However, the role and impact of ^18^F-FDG PET or ^18^F-FDG PET/CT is not as well established in SCLC.

A number of small nonrandomized prospective and retrospective studies have evaluated the impact of ^18^F-FDG PET or ^18^F-FDG PET/CT on the staging of SCLC. These studies showed that ^18^F-FDG PET or ^18^F-FDG PET/CT upstages 0% to 47% of limited-stage patients, but the results are inconsistent and many studies had significant bias.^16^, ^18^, ^19^, ^29^ Additional references are listed in Supplement 2. Furthermore and unlike in NSCLC studies, the rapid SCLC doubling time, coupled with the lack of need for precise anatomic histological staging to inform treatment decisions, has nearly always precluded histological validation of ^18^F-FDG PET or ^18^F-FDG PET/CT findings. The drive to integrate ^18^F-FDG PET/CT into routine practice in patients with SCLC is thus mainly based on indirect NSCLC evidence, limited studies in SCLC, and the appearance of SCLC lesions on ^18^F-FDG PET, typically exhibiting intense ^18^F-FDG activity.

Current practice guidelines recommend (the National Comprehensive Cancer Network^21^ and National Institute for Health and Care Excellence)^22^ or suggest (the American Society of Clinical Oncology, endorsing the American College of Chest Physicians guidelines)^23^ using ^18^F-FDG PET/CT for staging of patients with SCLC with or as an alternative to conventional imaging (European Society for Medical Oncology).^20^ However, there is limited evidence to support a change in therapy based on ^18^F-FDG PET/CT findings, as landmark trials that established chemoradiotherapy as standard limited-staged SCLC treatment were performed before the ^18^F-FDG PET/CT era.^24^ It is therefore likely that these trials inadvertently included patients with metastatic disease that was undetected with use of conventional imaging.

In our analysis, patients staged with ^18^F-FDG PET/CT in addition to conventional imaging had significantly smaller gross tumor volume, which could be partially due to more accurate tumor definition (e.g., ^18^F-FDG PET/CT is superior in differentiating collapsed lung from tumor and guiding nodal gross tumor volume definition). This led to lower radiotherapy doses delivered to normal tissues (lung, heart, and esophagus) and a lower incidence of late esophageal toxicity in ^18^F-FDG PET/CT–staged patients than in the conventional imaging group. A previous perspective study has highlighted the role of ^18^F-FDG PET in guiding selective nodal irradiation in patients with limited-stage SCLC.^30^ Prophylactic nodal irradiation was not allowed in the CONVERT trial. ^18^F-FDG PET/CT–staged patients were also less likely to have increased pretreatment serum LDH levels compared with patients staged with conventional imaging. This is explained by the inclusion of less bulky disease in the ^18^F-FDG PET/CT group. Furthermore, this could indicate that patients with metastatic disease were inadvertently included in the trial in the conventional imaging group. Nonetheless, survival outcomes were not different in patients staged with or without ^18^F-FDG PET/CT. These results suggest benefit from chemoradiotherapy in patients without macroscopic metastatic disease on conventional imaging, some of whom may harbor low-burden metastatic disease on ^18^F-FDG PET/CT. This is in keeping with recently reported studies demonstrating survival advantage of local ablative radiotherapy in oligometastatic NSCLC.^31^, ^32^ Evidence also supports the investigation of intensive radiotherapy in patients with extensive-stage SCLC and a limited number of extracranial extrathoracic metastases.^33^ We also report no significant difference in overall survival postradiological progression in patients staged with or without ^18^F-FDG PET/CT, providing indirect evidence that salvage therapies at the time of relapse were not different between study groups.

The findings of this study are important for a number of reasons. First, we provide indirect evidence that the improved survival reported in CONVERT is not due to a stage migration effect. Second, our findings suggest that conventional imaging could be acceptable to select patients with limited-stage SCLC for concurrent chemoradiotherapy. This has potential financial and logistical advantages, particularly in health care settings with limited access to a ^18^F-FDG PET/CT facility. A detailed economic analysis is beyond the scope of this study. Omission of ^18^F-FDG PET/CT could also shorten the staging pathway and time to initiation of therapy, which is important owing to the short SCLC tumor doubling time. The findings of this unplanned analysis should be confirmed in a prospective trial. The ongoing CALGB 30610/RTOG 0538 study (clinicaltrials.gov identifier: NCT00632853) may provide additional information on the optimal radiological staging of patients with limited-stage SCLC.

At the time the CONVERT trial protocol was developed, there was no consensus on the routine use of bone scintigraphy and ^18^F-FDG PET/CT for staging and treatment decisions in SCLC. The British Thoracic Society and the European Society of Medical Oncology guidelines did not mandate either as part of the staging investigations at the time.^20^, ^34^ For this reason, centers taking part in CONVERT were given the choice to use these staging modalities as per local practice. The trial protocol however specified that bone scintigraphy was to be performed if there was a specific clinical indication. Nonetheless, most patients in this study (57%) were staged with ^18^F-FDG PET/CT in addition to conventional imaging. However, the utilization of ^18^F-FDG PET/CT was variable in the eight countries recruiting to the CONVERT trial (41%–100%). In the conventional imaging group, only 15% of patients had bone scintigraphy, reflecting standard European and Canadian practice at the time.

Meta-analysis of small retrospective and nonrandomized prospective studies demonstrated the prognostic significance of pretreatment ^18^F-FDG PET volumetric and metabolic parameters in SCLC,^35^ but these results are inconsistent.^36^ Complete data on pretreatment ^18^F-FDG PET parameters in our study were unavailable. A separate exploratory analysis on the prognostic significance of ^18^F-FDG PET volumetric and metabolic parameters in patients recruited to CONVERT from UK centers is ongoing.

Study limitations include the exploratory nature of this analysis and the limited number of patients, particularly those staged by using conventional imaging. Although unknown confounders cannot be reliably excluded, it is noteworthy that baseline (gross tumor volume and pretreatment serum LDH level) and treatment (delivered chemotherapy cycles) characteristics were imbalanced, favoring ^18^F-FDG PET–staged patients. The CONVERT trial eligibility criteria (e.g., exclusion of patients with adverse biochemical features such as high LDH level) could have excluded a proportion of patients with subclinical metastases and influenced the study results. Data on the percentage of patients who were up-staged from limited disease to extensive disease or down-staged from extensive disease to limited disease on the basis of ^18^F-FDG PET/CT findings before randomization in CONVERT were unavailable. In the former scenario, most would have been offered palliative treatment. ^18^F-FDG PET/CT findings that influence treatment decisions should ideally be confirmed pathologically.^20^ Finally, this analysis does not address the role of ^18^F-FDG PET/CT to guide the radiation oncologist in the definition of the gross tumor volume and the impact on radiation portals.

In CONVERT, survival outcomes were not significantly different in patients staged with or without ^18^F-FDG PET/CT. However, this analysis cannot support the use or omission of ^18^F-FDG PET/CT because of study limitations.
