# Supplementary material for: 18F-Fludeoxyglucose PET/CT in SCLC: Analysis of the CONVERT Randomized Controlled Trial
Source: J Thorac Oncol. 2019 Jul;14(7):1296–305. doi: 10.1016/j.jtho.2019.03.023 (PMC6616906; doi:10.1016/j.jtho.2019.03.023)
Supplement: Supplement 2 [file mmc2.docx]

**SUPPLEMENTARY MATERIAL**

**ADDITIONAL STATISTICAL DETAILS**

*Univariate and multivariate analyses****:*** Data on the following covariates were collected for univariate and multivariate analyses: Eastern Cooperative Oncology Group performance score, age, ^18^F-FDG PET/CT staging, gross tumour volume, percentage of total dose received by heart (heart-dose), volume of the lung receiving ≥20Gy (lung V20), alkaline phosphatase, hyponatremia, lactate dehydrogenase, smoking status, weight loss, forced expiratory volume in 1 sec percent predicted (FEV1 %P), KCO percent predicted (KCO %P), and UICC/ AJCC stage. For each variable an assessment of the proportional hazards assumption was done via Schonenfeld residuals plots.


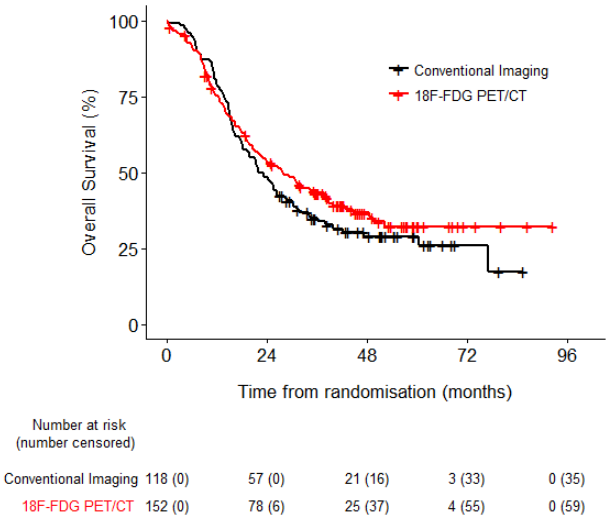

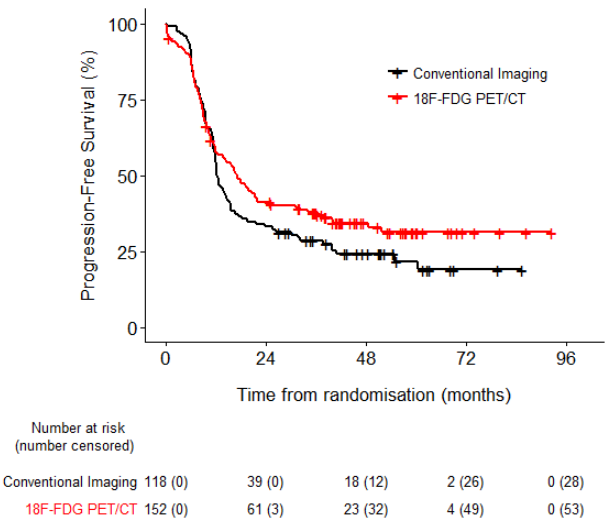

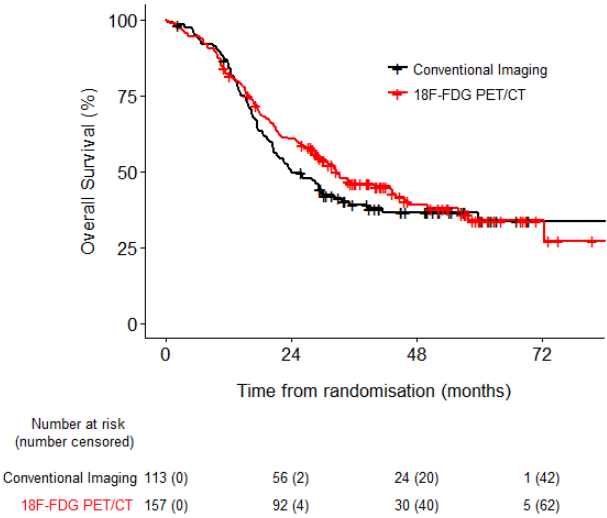

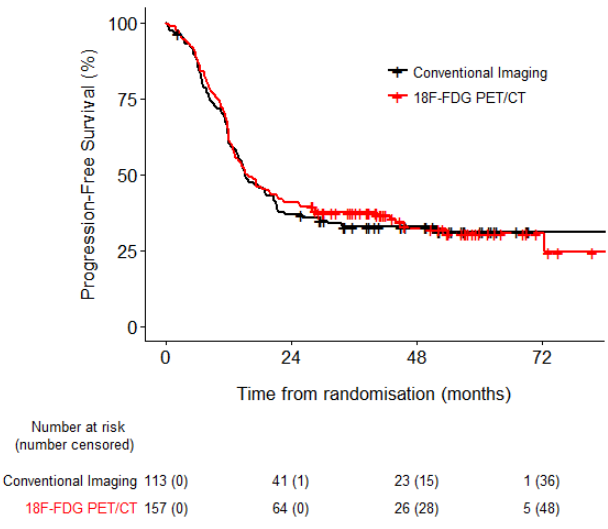


**A**

**B**

**C**

**D**

**H.R. 0·86 (95% CI 0·64-1·16); p=0·331**

**H.R. 0·96 (95% CI 0·72-1·29); p=0·800**

**H.R. 0·88 (95% CI 0·64-1·20); p=0·414**

**H.R. 0·80 (95% CI 0·60-1·07); p=0·131**

**Supplementary Fig. 1**

|  | **^18^F-FDG PET/CT and conventional imaging** | **Conventional imaging** |
| --- | --- | --- |
| UK | 132 (41%) | 188 (59%) |
| France | 78 (76%) | 24 (24%) |
| Canada | 28 (72%) | 11 (28%) |
| Spain | 22 (81%) | 5 (19%) |
| Belgium | 23 (96%) | 1 (4%) |
| Slovenia | 13 (100%) | 0 (0%) |
| Netherlands | 9 (100%) | 0 (0%) |
| Poland | 4 (67%) | 2 (33) |

**Supplementary Table 1:** ^18^F-FDG PET/CT uptake across countries

**Supplementary Table 2:** Univariate and multivariate progression-free survival analysis

|  | **Patients** | **Univariate analysis** | | **Multivariate analysis** | |
| --- | --- | --- | --- | --- | --- |
|  | **Events/*n*** | **HR (95% CI)** | **p-value** | **HR (95% CI)** | ***P*-value** |
| ECOG PS  1 or 2 v 0 | 370/540 | 1·28 (1·04 – 1·57) | 0·019 | 1·12 (0·87 – 1·44) | 0·366 |
| Age | 370/540 | 1·00 (0·99 – 1·02) | 0·499 | 1·00 (0·98 – 1·01) | 0·603 |
| log(GTV) | 318/480 | 1·34 (1·19 – 1.51) | <0·001 | 1·26 (1·09 – 1.45) | **0·002** |
| Heart-dose  (%) | 312/469 | 1·89 (0·30 – 11·86) | 0·498 | 1·00 (0·98 – 1·02) | 0·936 |
| V20 Lung  (%) | 330/493 | 1·02 (1·01 – 1·04) | 0·003 | 1·00 (0·98 – 1·02) | 0·970 |
| ALP >1.5xULN  Yes v No | 370/540 | 1·18 (0·56 – 2·48) | 0·673 | 2·15 (0.63 – 7·26) | 0·219 |
| Hyponatremia  Yes v No | 370/540 | 0·92 (0·72 – 1·19) | 0·541 | 1·03 (0·74 – 1·44) | 0·858 |
| LDH >ULN  Yes v No | 370/540 | 0·85 (0·67 – 1·08) | 0·175 | 0·95 (0·70 – 1·27) | 0·720 |
| Smoking  ex v never  current v never | 370/540 | 0·65 (0·27 – 1·58)  0·70 (0·29 – 1·71) | 0·342  0·437 | 0·83 (0·25 – 2·70)  0·88 (0·27 – 2·88) | 0·753  0·833 |
| Weight Loss >10%  Yes v No | 348/500 | 1·63 (1·03 – 2·59) | 0·037 | 1·81 (1.03 – 3·16) | **0·039** |
| FEV1 % predicted | 351/515 | 0·99 (0·99 – 1·00) | 0·012 | 0·99 (0·99 – 1·00) | 0·092 |
| KCO % predicted | 351/515 | 0·99 (0·99 – 1·00) | 0·767 | 1·00 (0·99 – 1·01) | 0·979 |
| Disease stage  III v I or II | 354/509 | 1·67 (1·23 – 2·27) | <0·001 | 1·41 (0·97 – 2·05) | 0·069 |
| ^18^F-FDG PET/CT  Yes v No | 370/540 | 0·87 (0·71 – 1·07) | 0·198 | 0·92 (0·72 – 1·18) | 0·527 |

ECOG PS – Eastern Cooperative Oncology Group Performance Status; GTV – Gross Tumour Volume; ALP – Alkaline Phosphatase; ULN – Upper Limit of Normal; LDH – Lactate Dehydrogenase; HR – Hazard Ratio; CI – Confidence Interval.

| **Progression Site** | **^18^F-FDG PET/CT and conventional imaging (n=309, events=204)^1^** | **Conventional imaging**  **(n=231, events=165)^1^** |
| --- | --- | --- |
| Local (%) | 68 (21%) | 51 (20%) |
| Nodal (%)  Hilar  Mediastinal  Supraclavicular | 54 (17%)  21  26  7 | 53 (20%)  17  24  12 |
| Distant (%)  Contralateral lung  Liver  Bone  Brain  Others | 200 (62%)  11  45  29  55  60 | 156 (60%)  8  38  21  32  57 |

**Supplementary Table 3:** Sites of tumor progression in both study groups

^1^: In patients with more than one site of tumour progression recorded on the case report forms; these were registered separately in each category

**Supplementary Table 4:** Delivered radiotherapy according to treatment arm in both study groups

|  | **^18^F-FDG PET/CT and conventional imaging (n=309)** | **Conventional imaging**  **(n=231)** | ***P*-value** |
| --- | --- | --- | --- |
| **Radiotherapy fractions delivered (once-daily/ twice-daily)** | | | |
| <33/<30 | 49  (28/21) | 42  (23/18) | 0·397 |
| 33/30 | 231  (106/125) | 174  (86/87) |  |
| >33/>30 | 2  (1/1) | 0  (0/0) |  |
| **Radiotherapy dose delivered (once-daily/ twice-daily)** | | | |
| <28 Gy (%) | 1  (1/0) | 0  (0/0) | 0·187 |
| <60/ <44 Gy (%) | 11  (11/0) | 17  (15/2) |  |
| ≥60/ ≥44 Gy (%) | 270  (123/147) | 197  (94/103) |  |

**Supplementary Table 5:** Dosimetric radiotherapy parameters in both study groups

| **Radiotherapy parameter** | **^18^F-FDG PET/CT and conventional imaging (n=309)** | **Conventional imaging**  **(n=231)** | ***P*-value** |
| --- | --- | --- | --- |
| Minimum PTV dose (Gy)  median (range) | 42 (2-66) | 43 (4-63) | 0·870 |
| V5 lung (%)  median (range) | 57 (7-99) | 60 (20-89) | **0·009** |
| V20 lung (%)  median (range) | 25 (1-100) | 26 (8-38) | 0·067 |
| Mean lung dose (Gy)  median (range) | 14 (4-29) | 15 (3-23) | **0·018** |
| Spinal cord max. dose (Gy)  median (range) | 36 (1-53) | 38 (2-49) | 0·052 |
| Dose to heart (%)  median (range) | 1 (0-29) | 2 (0-45) | **0·006** |
| Esophagus max. dose (Gy)  median (range) | 45 (1-72) | 48 (10-71) | **0·015** |
| Length esophagus Receiving≥40 Gy (cm)  median (range) | 9 (0-21) | 10 (0-24) | **0·001** |
| Length esophagus treated in 95% isodose (cm)  median (range) | 7 (0-24) | 8 (0-19) | **0·002** |
| V35 esophagus (%)  median (range) | 34 (0-95) | 39 (0-95) | **0·003** |

**Supplementary Table 6:** Comparison of acute toxicity between the two groups

| **Toxicity** | **Grade** | **^18^F-FDG PET/CT and conventional imaging (n=309)** | **Conventional imaging**  **(n=231)** | ***P*-value** |
| --- | --- | --- | --- | --- |
| Esophagitis (%) | 0  1  2  3  4 | 90 (29%)  65 (21%)  104 (34%)  49 (16%)  1 (<1%) | 63 (27%)  40 (17%)  82 (35%)  46 (20%)  0 (0%) | 0·523 |
| Pneumonitis (%) | 0  1  2 | 291 (94%)  15 (5%)  3 (1%) | 213 (92%)  13 (6%)  5 (2%) | 0·478 |
| Dermatitis (%) | 0  1  2 | 234 (76%)  61 (20%)  14 (5%) | 174 (75%)  42 (18%)  15 (6%) | 0·571 |
| Neutropenia (%) | 0  1  2  3  4 | 54 (17%)  20 (6%)  28 (9%)  77 (25%)  130 (42%) | 34 (15%)  18 (8%)  20 (9%)  59 (26%)  100 (43%) | 0·905 |

**Supplementary Table 7:** Comparison of late toxicity between the two groups

| **Toxicity** | **Grade** | **^18^F-FDG PET/CT and conventional imaging (n=309)** | **Conventional imaging**  **(n=231)** | ***P*-value** |
| --- | --- | --- | --- | --- |
| Esophagitis (%) | 0  1  2  3 | 277 (90%)  15 (5%)  12 (4%)  5 (2%) | 188 (81%)  27 (12%)  14 (6%)  2 (1%) | **0·012** |
| Pneumonitis (%) | 0  1  2  3  4 | 215 (70%)  53 (17%)  33 (11%)  6 (2%)  2 (1%) | 172 (74%)  40 (17%)  15 (6%)  4 (2%)  0 (0%) | 0·334 |
| Dermatitis (%) | 0  1  2 | 294 (95%)  14 (5%)  1 (<1%) | 214 (93%)  15 (6%)  2 (1%) | 0·420 |
| Myelitis (%) | 0  1 | 303 (98%)  6 (2%) | 228 (99%)  3 (1%) | 0·812 |
| Pulmonary fibrosis (%) | 0  1  2  3 | 189 (61%)  93 (30%)  25 (8%)  2 (1%) | 121 (52%)  94 (41%)  13 (6%)  3 (1%) | 0·050 |
| Esophageal fistula (%) | 0  >1 | 309 (100%)  0 (0%) | 231 (100%)  0 (0%) | N/A |
| Esophageal stricture (%) | 0  1  2  3 | 300 (97%)  6 (2%)  2 (1%)  1 (<1%) | 225 (97%)  3 (1%)  2 (1%)  1 (<1%) | 0·928 |

**ADDITIONAL REFERENCES ON THE ROLE OF STAGING ^18^F-FDG PET IN SMALL-CELL LUNG CANCER**

1. Arslan N, Tuncel M, Kuzhan O, et al. Evaluation of outcome prediction and disease extension by quantitative 2-deoxy-2-[18F] fluoro-D-glucose with positron emission tomography in patients with small cell lung cancer. Annals of nuclear medicine 2011; 25(6): 406-13.

2. Azad A, Chionh F, Scott AM, et al. High impact of 18F-FDG-PET on management and prognostic stratification of newly diagnosed small cell lung cancer. Molecular imaging and biology : MIB : the official publication of the Academy of Molecular Imaging 2010; 12(4): 443-51.

3. Blum R, MacManus MP, Rischin D, Michael M, Ball D, Hicks RJ. Impact of positron emission tomography on the management of patients with small-cell lung cancer: preliminary experience. American journal of clinical oncology 2004; 27(2): 164-71.

4. Brink I, Schumacher T, Mix M, et al. Impact of [18F]FDG-PET on the primary staging of small-cell lung cancer. European journal of nuclear medicine and molecular imaging 2004; 31(12): 1614-20.

5. Chin R, Jr., McCain TW, Miller AA, et al. Whole body FDG-PET for the evaluation and staging of small cell lung cancer: a preliminary study. Lung cancer 2002; 37(1): 1-6.

6. Kamel EM, Zwahlen D, Wyss MT, Stumpe KD, von Schulthess GK, Steinert HC. Whole-body (18)F-FDG PET improves the management of patients with small cell lung cancer. Journal of nuclear medicine : official publication, Society of Nuclear Medicine 2003; 44(12): 1911-7.

7. Kut V, Spies W, Spies S, Gooding W, Argiris A. Staging and monitoring of small cell lung cancer using [18F]fluoro-2-deoxy-D-glucose-positron emission tomography (FDG-PET). American journal of clinical oncology 2007; 30(1): 45-50.

8. Niho S, Fujii H, Murakami K, et al. Detection of unsuspected distant metastases and/or regional nodes by FDG-PET [corrected] scan in apparent limited-disease small-cell lung cancer. Lung cancer 2007; 57(3): 328-33.

9. Oh JR, Seo JH, Chong A, et al. Whole-body metabolic tumour volume of 18F-FDG PET/CT improves the prediction of prognosis in small cell lung cancer. European journal of nuclear medicine and molecular imaging 2012; 39(6): 925-35.

10. Pandit N, Gonen M, Krug L, Larson SM. Prognostic value of [18F]FDG-PET imaging in small cell lung cancer. European journal of nuclear medicine and molecular imaging 2003; 30(1): 78-84.

11. Saima R, Humayun B, Khalid NI. Triage of Limited Versus Extensive Disease on (18)F-FDG PET/CT Scan in Small Cell lung Cancer. Asia Oceania journal of nuclear medicine & biology 2017; 5(2): 109-13.

12. Schumacher T, Brink I, Mix M, et al. FDG-PET imaging for the staging and follow-up of small cell lung cancer. European journal of nuclear medicine 2001; 28(4): 483-8.

13. Shen YY, Shiau YC, Wang JJ, Ho ST, Kao CH. Whole-body 18F-2-deoxyglucose positron emission tomography in primary staging small cell lung cancer. Anticancer research 2002; 22(2b): 1257-64.
